# Slow Is Also Fast: Feedback Delay Affects Anxiety and Outcome Evaluation

**DOI:** 10.3389/fnhum.2018.00020

**Published:** 2018-01-25

**Authors:** Xukai Zhang, Yi Lei, Hang Yin, Peng Li, Hong Li

**Affiliations:** ^1^Brain Function and Psychological Science Research Center, Shenzhen University, Shenzhen, China; ^2^Research Center of Brain and Cognitive Neuroscience, Liaoning Normal University, Dalian, China; ^3^Shenzhen Key Laboratory of Affective and Social Cognitive Science, Shenzhen University, Shenzhen, China; ^4^Center for Emotion and Brain, Shenzhen Institute of Neuroscience, Shenzhen, China

**Keywords:** outcome evaluation, learning, anxiety, feedback delay, reward positivity

## Abstract

Performance-related feedback plays an important role in improving human being’s adaptive behavior. Using event-related potentials (ERPs), previous studies have associated a particular component, i.e., reward positivity (RewP), with outcome evaluation processing and found that this component was affected by waiting time before outcome evaluation. Prior research has also suggested that anxious individuals are more prone to detecting threats and susceptible to negative emotions, and show different patterns of brain activity in outcome evaluation. It is quite common that a decision-maker cannot receive feedback immediately; however, few studies have focused on the processing of delayed feedback, especially in subjects who exhibit trait anxiety. In this study, we recruited two groups of subjects with different trait anxiety levels and recorded ERPs when they conducted a time-estimation task with short (0.6–1 s) or long delayed (4–5 s) feedback. The ERP results during the cue phase showed that long waiting cues elicited more negative-going feedback-related negativity (FRN)-like component than short waiting cues in the high trait anxiety (HTA) group. More importantly, the two groups showed different patterns of ERP in the feedback condition. In the low trait anxiety (LTA) group, more positive-going RewP was found in the short-delayed than in the long-delayed condition. In contrast, no difference was found in the HTA group. This pattern may reflect the hyperactivity of the reward systems of HTA individuals in uncertain environments (e.g., the long-delay condition) compared with LTA individuals. Our results provide a direction for future research on the neural mechanisms of reinforcement learning and anxiety.

## Introduction

Learning from the environment can help people improve their behavior; performance-related feedback therefore plays a key role in adapting to a changing environment. Specially, according to the reinforcement learning theory, a decision-maker has a high chance to repeat behaviors which provided reward feedbacks and to avoid behaviors which lead to non-reward feedbacks before. The motivation of pursuing reward drives decision-makers select actions associated with high expectation of reward and feedback backward updates corresponding reward expectation of certain actions (Schultz et al., 1997). The so-called reward prediction error refers to the difference between current outcome and expected outcome (Schultz et al., 1997). Researchers found that a particular event-related brain potential (ERP) component, feedback-related negativity (FRN), was highly related to reward prediction error during outcome processing (Holroyd and Coles, 2002). FRN is a frontocentral negativity deflection maximal at approximately 200–300 ms following the presentation of feedback (Miltner et al., 1997; Holroyd and Coles, 2002; Li et al., 2016). A more negative-going FRN is often observed in the processing of negative feedback, compared to that of positive feedback (Yeung and Sanfey, 2004; Ferdinand et al., 2012). A large body of research indicates that FRN reflects decreased phase of dopaminergic signal in the basal ganglia and that the generation of this potential occurs in the anterior cingulate cortex (ACC; Bellebaum and Daum, 2008; Holroyd et al., 2009; Hauser et al., 2014; Holroyd and Umemoto, 2016; but see Foti et al., 2011; Becker et al., 2014; Sambrook and Goslin, 2016).

Early on, the FRN was mainly associated with a large negative waveform elicited by losses (negative feedback) and absent for rewards (e.g., Holroyd and Coles, 2002; Hajcak et al., 2006). According to the influential reinforcement learning error-related negativity (RL_ERN) theory, the midbrain dopamine system is a key component for detecting and monitoring whether the actual outcomes match the expectations, then sending corresponding dopaminergic signals to the ACC, where a larger negative waveform is generated (Holroyd and Coles, 2002). However, this theory was not supported by several following studies. Oliveira et al. (2007) argued that the ACC activity was increased due to violations in expectancy, irrespective of outcome valence. This finding was confirmed by some other studies (Alexander and Brown, 2010; Ferdinand et al., 2012). With the development of the theory, Holroyd et al. (2008) proposed a new idea based on their original RL-ERN theory. They posited that the FRN was mainly driven by positive feedback: unexpected positive feedback increased the phase change in dopamine, which inhibited the conflict signal in ACC and thus canceled out the amplitude of the N200. Substantial evidence indicated greater modulation of FRN by correct feedback than by error feedback (Potts et al., 2006; Eppinger et al., 2008; Hewig et al., 2008; Holroyd and Coles, 2008; Heydari and Holroyd, 2016). The potential positive component elicited by reward feedback was renamed reward positivity (RewP) and was calculated by the difference wave of the positive feedback subtracted from the negative feedback (Baker and Holroyd, 2011).

Most recent studies have mainly focused on immediate feedback processing; however, in daily life, it is quite common that a person cannot receive feedback or reward immediately after decision-making. In recent years, many studies have investigated how delayed rewards influence decision-making (Green et al., 1994; Wittmann et al., 2007). Some researchers have conducted single-unit recording studies and indicated that the dopamine signal dynamically varied in different waiting time conditions, with stronger neuronal firing with an immediate reward compared to a delayed reward (Roesch et al., 2007). Subsequent studies have shown that the striatum and hippocampus have been involved in outcome processing for immediate and delayed rewards, respectively (Foerde and Shohamy, 2011). The variance in the activity of the reward system when the reward is delayed could be due to a shift from nondeclarative to declarative learning. Therefore, it would be interesting to know whether delayed feedback could also improve reinforcement learning in terms of behavioral adjustment.

Recently, several groups of researchers began to examine delayed feedback in EEG experiments. The purpose of the initial study was to explore whether temporal delay could affect RewP component elicited by monetary feedback in a gambling task (Weinberg et al., 2012). Their results showed that the RewP^1^ was negligible when the delay was several seconds long. Subsequently, further research explored the linear effects of delayed time on outcome evaluation and tested whether the learning of optimal choice will be affected by different waiting time in a feedback learning task (Peterburs et al., 2016). Their results showed that the RewP decreased gradually with increased delay time, but no effect of time was found on behavioral learning. Moreover, Weismuller and Bellebaum (2016) used a probabilistic learning task to investigate whether the delayed feedback was sensitive to expectancy, and found the RewP was significantly more positive-going for unexpected compared to expected feedback in both of immediate and delayed feedback condition. More recently, Arbel et al. (2017) reported that the RewP component was only observed in immediate feedback condition. In our previous study, participants were asked to choose which balloon had money before making the selection; they would then see a cue that represented a long or short wait for the choice’s outcome. Compared to the findings of other groups, we found that waiting time did not affect the FRN component (Wang et al., 2014). These inconsistent findings could be caused by two possible factors. First, most of these studies utilized random monetary feedback regardless of participants’ actual performance. Although previous studies used a probabilistic learning task, the learning may only have happened in the beginning phase because participants could maintain the advantageous choice they learned before in the last phase. Second, none of these studies have looked at individual differences in the processing of waiting time. A longer waiting time could cause greater uncertainty, which in turn could influence participants’ anxiety (Maner and Schmidt, 2006).

A large number of studies have shown that individual differences in factors such as stress, depression and anxiety influence the outcome evaluation process in decision-making (Foti and Hajcak, 2009; Gu et al., 2010a,b; Li et al., 2015). One study found that anxiety could enhance the perception of threats (Maner and Schmidt, 2006). High-anxiety individuals also showed more negative exception bias (Eisenberg et al., 1998; Wray and Stone, 2005; Maner and Schmidt, 2006). Notably, most of these findings came from self-reports data. Gu et al. (2010a) used ERP to investigate the relationship between anxiety and outcome evaluation; they found that the RewP in the high trait anxiety (HTA) group was more positive-going than that in low trait anxiety (LTA) groups in ambiguous conditions, and they proposed that ambiguity makes feedback more threatening. The HTA individuals also showed less positive-going RewP than LTA individuals in negative conditions, confirming the negative bias of anxious individuals. While waiting for feedback can itself cause uncertainty, individual differences are a major cause of inconsistency in perceived uncertainty (Hirsh et al., 2012). Furthermore, neuroimaging studies have discussed the function of the hippocampus in anxiety and memory (for a review see Bannerman et al., 2004). Recent findings provide evidence that the dorsal hippocampus is not immune to anxiety and that trait anxiety shows more activity in the dorsal hippocampus than state anxiety, while the subregions may overlap with the memory function area (Satpute et al., 2012).

Based on the characteristics of anxious individuals described above, the purpose of this study was, first, to explore whether the length of waiting time for feedback in a trial-and-error task would affect learning, and second, to investigate whether the manipulation of waiting time would affect the outcome evaluation of trait anxiety individuals. Data analysis mainly focused on the behavioral adjustment data and the RewP amplitude. We hypothesized that more positive-going RewP would be observed in the short delay condition than that in the long delay condition in LTA individuals, but not in HTA individuals because the long waiting time would cause higher anxiety degrees for HTA than LTA groups (Hirsh et al., 2012). We also predicted that the adjustment of behavior after the long delay would be better than short delay due to the effect of waiting time on learning (Butler et al., 2007; Guzmán-Muñoz and Johnson, 2008; Metcalfe et al., 2009). Furthermore, we examined whether different brain activities could be observed in the HTA and LTA groups even in the cue phase when they first learn how long they will have to wait.

## Materials and Methods

### Participants

A total of 739 undergraduate students were tested using the Chinese version of the State-Trait Anxiety Inventory (STAI; Spielberger et al., 1983). Seventeen students (13 females, age = 20.06 ± 1.09) whose scores were in the top 25% were randomly selected as the HTA subjects (pre-STAI scores = 57.35 ± 4.39), while another 17 students (13 females, age = 20.53 ± 1.70) whose scores were in the lowest 25% were randomly recruited as the LTA subjects (pre-STAI scores = 30.06 ± 2.05). The trait anxiety scores in the two groups were significantly different, *t*_(32)_ = 23.11, *p* < 0.001. This result was confirmed by a STAI retest a week later when the formal experiment was conducted, *t*_(32)_ = 12.79, *p* < 0.001. The post-STAI scores of HTA subjects (53.76 ± 6.68) was significantly larger than that of the LTA group (29.53 ± 4.06). All participants had normal or corrected-to-normal vision and normal audition, and none had a neurological or psychological history. Informed written consent was obtained before the experiment, which was approved by the Ethics Committee of Liaoning Normal University. Each subject received 38–42 yuan based on the accuracy of the experimental task.

### Experimental Procedure

**Figure 1:**
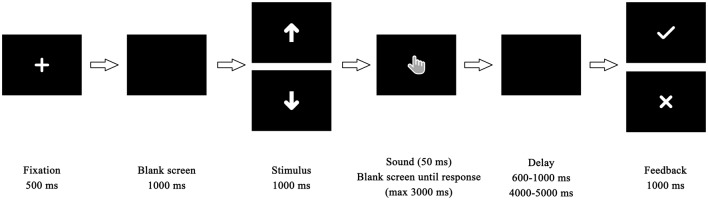
Time course of stimulus presentation in the time estimation task.

Before the experiment, the participants were told they would complete a time estimation task (Miltner et al., 1997). The task was modified based on the classic time estimation task. Participants were to change or maintain current behavior in response to feedback to maintain the right perception of the duration of 1 s. In each trial (Figure 1), a fixation screen was shown for 500 ms, then replaced by a blank screen for 1000 ms. Afterward, an arrow indicating how long the participant would wait before final feedback was presented for 1000 ms. There were two different arrow cues with different orientations, which corresponded to long delay or short delay. The meanings of the two types of cues were counterbalanced between participants. Following the cue, the participants heard a sound (1500 Hz, 50 dB, lasting 50 ms) that indicated the beginning of the estimation. Participants were asked to estimate 1 s and to press the space button on the keyboard as soon as they believed that a second of time had passed. The duration between participants’ responses and feedback stimuli was set randomly between 4000 ms and 5000 ms in the long-delay condition and between 600 ms and 1000 ms in the short-delay condition. Finally, feedback appeared in white on a black background and lasted for 1000 ms. If participants’ estimation was within the required time window (900–1100 ms), they received positive feedback indicated by a black “√” mark; otherwise, the feedback was an “X” mark. The inter-trial interval was set randomly between 1000 ms and 1500 ms.

The initial time window was set as 900 ms to 1100 ms. If a participant’s reaction time (RT) was in this interval, their estimation was considered to be correct in that trial. Conversely, if their RT was not in this interval, they received the error feedback. The preset time window was adjusted according to the participants’ performance trial by trial: 10 ms was added if they responded incorrectly and 10 ms was subtracted if they respond correctly. All of the participants received about 50% correct and 50% incorrect feedback overall based on the algorithm. The whole experiment consisted of 120 trials in the long-delay condition and 120 trials in short-delay condition.

The self-report questionnaire was completed when participants finished the experiment. In this questionnaire, the participants were asked to rate how much attention they paid to the outcome when the waiting time was long or short (1 = “ignored outcomes”; 7 = “paid close attention”), how they felt when they responded correctly in the long-delay condition and short-delay condition (1 = “very unhappy”; 7 = “very happy”), and how they felt when they responded incorrectly in the long-delay condition and short-delay condition (1 = “very unhappy”; 7 = “very happy”).

### Data Recording and Analysis

The present study was a 2 (waiting time: long or short) × 2 (valence: correct or incorrect) × 2 (trait anxiety: HTA or LTA) design. In order to investigate the effect of valence and waiting time on behavior, we calculated the absolute changes in RT (∆RT), meaning the absolute values of the different RTs between the current trial and the next trial (Holroyd and Krigolson, 2007; Li et al., 2016). Note that ∆RT is better than raw RT to indicate participants’ behavioral adjustment because they could increase or decrease their RT after receiving negative feedback in such a time-estimation task. The ∆RT data was submitted to a 2 × 2 × 2 repeated measures analysis of variance (ANOVA) with trait anxiety (LTA or HTA) as the between-subject factor and with waiting time (long or short) and valence (correct or incorrect) as within-subject factors.

Continuous EEG data were recorded with a 64-channel system (eego, eemagine, Germany) and sampled at 500 Hz. The CPz was used as a reference online, and the left and right mastoids were digitally converted to average as re-references offline. Vertical electrooculograms were obtained via facial electrodes located above and below the left eye. Horizontal electrooculograms were obtained via facial electrodes placed at the outer canthi of the eyes. Impedances of all electrodes was kept under 15 kΩ. EEG data were analyzed by BrainVision Analyzer 2.0 software (Brain Products, Germany). A 0.1–20 Hz passband filter was applied to EEG data offline. Eye blink and ocular artifacts were corrected using independent component analysis (Lee et al., 1999). The segments ranged from −200 ms before the cue/feedback onset to 800 ms after the cue/feedback was presented. Before the cue/feedback stimulus onset, a −200 to 0 ms time range was used as a baseline. All of the periods in which the maximal amplitude was over ±80 μV were rejected as artifacts. Less than 5% of trials were rejected in each condition. Finally, the EEG data were averaged in each condition for further analysis in both the cue phase and feedback phase separately.

The RewP amplitude was calculated by the difference waves at electrode Fz between negative feedback and positive feedback (Holroyd and Krigolson, 2007). The electrode Fz was selected because RewP reached the maximum peak at this site (Li et al., 2010, 2011; Wang et al., 2014). The RewP amplitude was measured by the mean amplitude in the 250–300 ms time window following feedback onset (Yeung et al., 2005; Yu and Zhou, 2006). The FRN-like in cue phase was measured by the mean amplitude in the 240–340 ms time window following cue onset. Additionally, the mean amplitudes of P300 were computed in a 340–440 ms time window at the electrode Pz where it peaks for feedback phase and cue phase respectively. The reason for analyzing this component is to investigate whether the amplitude of the FRN was confounded by P300 because of the general concern of component overlapping in this field (Gu et al., 2010a; Foti et al., 2011; Sambrook and Goslin, 2015). The FRN-like was submitted to a 2 (cue: short, long) × 2 (trait anxiety: high, low) repeated measures ANOVA for the cue phase and the RewP was submitted to 2 (waiting time: long, short) × 2 (trait anxiety: high, low) two-way ANOVA for the feedback phase. In addition, P300 amplitudes were submitted to a 2 (cue: short, long) × 2 (trait anxiety: high, low) repeated measures ANOVA for cue phase and 2 (waiting time: long, short) × 2 (valence: correct, incorrect) × 2 (trait anxiety: high, low) repeated measures ANOVA for the feedback phase. Greenhouse-Geisser corrections were used where necessary.

## Results

### Behavioral Results

**Figure 2:**
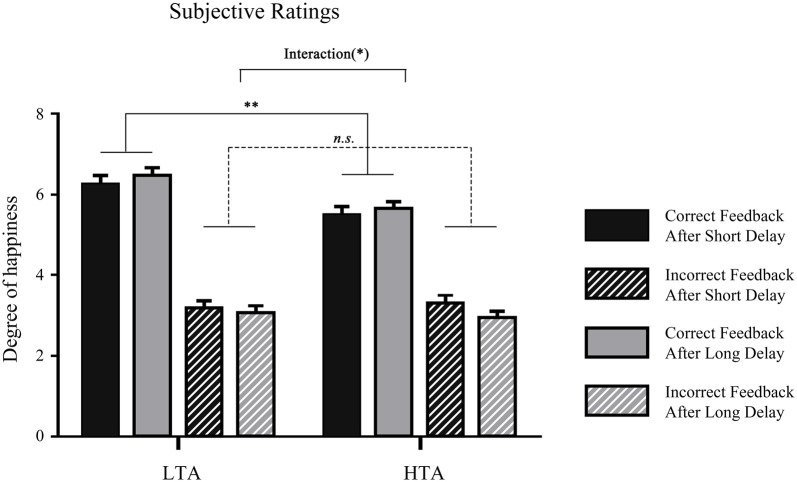
Mean degree of happiness in each waiting time condition in the two groups. **p < 0.01, *p < 0.05.

To examine whether attention was affected by the waiting time in two groups, the subjective ratings of attention in post-experiment questionnaire were submitted to a 2 (waiting time: long, short) × 2 (trait anxiety: high, low) repeated measures ANOVA. The results showed that the main effect of waiting time on attention did not reach significance, *F*_(1,32)_ = 0.019, *p* = 0.891, $\eta_{\text{p}}^{2}$ = 0.001. The interaction effect between waiting time and groups was not significant either, *F*_(1,32)_ = 0.93, *p* = 0.342, $\eta_{\text{p}}^{2}$ = 0.03. Moreover, the rating scores of feelings of happiness in the four different conditions: (a) waiting a long time for correct feedback; (b) waiting a long time for error feedback; (c) waiting a short time for correct feedback; and (d) waiting a short time for correct feedback, were submitted to a 2 (waiting time: long, short) × 2 (valence: correct, incorrect) × 2 (trait anxiety: high, low) repeated measures ANOVA, and the main effect feedback valence was significant, *F*_(1,32)_ = 278.24, *p* < 0.001, $\eta_{\text{p}}^{2}$ = 0.90, indicating that people felt happier when they received correct feedback than when they received incorrect feedback. Additionally, the interaction of trait anxiety and feedback valence was significant, *F*_(1,32)_ = 5.45, *p* = 0.026, $\eta_{\text{p}}^{2}$ = 0.15; pairwise comparison showed that the LTA group felt happier than the HTA group in the correct feedback condition (*p* = 0.003; Figure 2).

**Figure 3:**
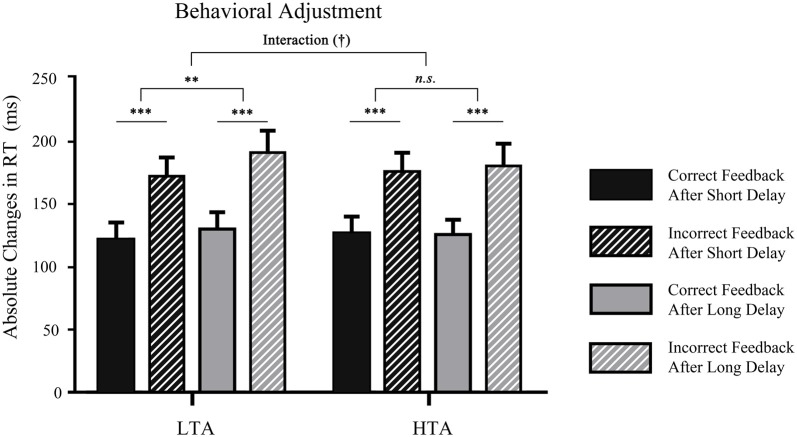
Mean time estimation adjustment in each waiting time condition in the two groups. ***p < 0.001, **p < 0.01. ^†^Means marginal significance.

To determine whether the delay and feedback type influenced subsequent behavior in different trait anxiety groups, the ∆RT was analyzed by using a 2 (waiting time: long, short) × 2 (valence: correct, incorrect) × 2 (trait anxiety: high, low) three-way ANOVA (see Figure 3). The main effect of waiting time was significant, *F*_(1,32)_ = 4.49, *p* < 0.05, $\eta_{\text{p}}^{2}$ = 0.12. The ∆RT in the short-delay condition (*M* = 155.92, *SD* = 10.73) was smaller than that in the long-delay condition (*M* = 148.87, *SD* = 9.87). The main effect of outcome also reached significance, *F*_(1,32)_ = 129.84, *p* < 0.001, $\eta_{\text{p}}^{2}$ = 0.80; prior error outcomes promoted change for the next trials (*M* = 179.46, *SD* = 11.32) and led to a larger time adjustment than prior correct outcomes (*M* = 125.33, *SD* = 9.50). It is noteworthy that the interaction between trait anxiety and waiting time was marginally significant (*p* = 0.072). A larger absolute response time in the long-delay condition was found only in LTA individuals (*p* = 0.008), not in HTA individuals (*p* = 0.855). No other significant effect was found (all *p* > 0.05).

### Electrophysiological Results

#### Cue Phase

**Figure 4:**
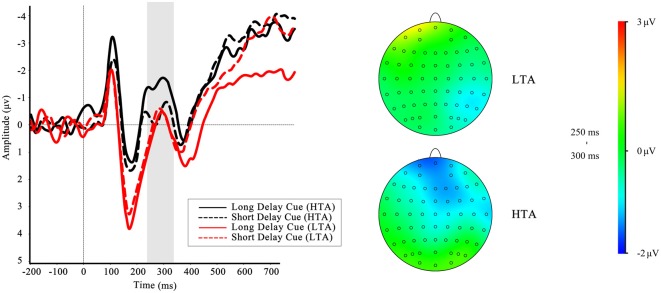
Cue-locked event-related potential (ERP) waveforms at Fz comparing long- and short-delayed cues in high trait anxiety (HTA) and low trait anxiety (LTA) groups, along with different waveforms and topographic maps for long- and short-delayed cues in HTA and LTA groups in time range of feedback-related negativity (FRN). Gray shaded area shows the 240–340 ms analysis window in which the FRN-like was quantified.

To investigate whether the brain activities of the HTA and LTA groups could differ even in the cue phase, we analyzed the FRN-like component according to one of our previous studies (Wang et al., 2016). The results showed a significant interaction effect between group and cue type on FRN-like amplitude, *F*_(1,32)_ = 4.66, *p* = 0.039, $\eta_{\text{p}}^{2}$ = 0.13. Simple effect analysis revealed that the effect of waiting time was significant in the HTA group, *F*_(1,32)_ = 7.21, *p* = 0.011, with more negative-going FRN-like amplitude for long-delay cues than short-delay cues in the HTA group, but not in the LTA group, *F*_(1,32)_ = 0.13, *p* = 0.717. The grand-average ERP waveforms for cues in the different trait anxiety groups are presented in Figure 4. The main effects of waiting time (*F*_(1,32)_ = 2.69, *p* = 0.111) and trait anxiety (*F*_(1,32)_ = 0.29, *p* = 0.60) were not significant. The same measures were used for P300, but no significant effects were found.

#### Feedback Phase

**Figure 5:**
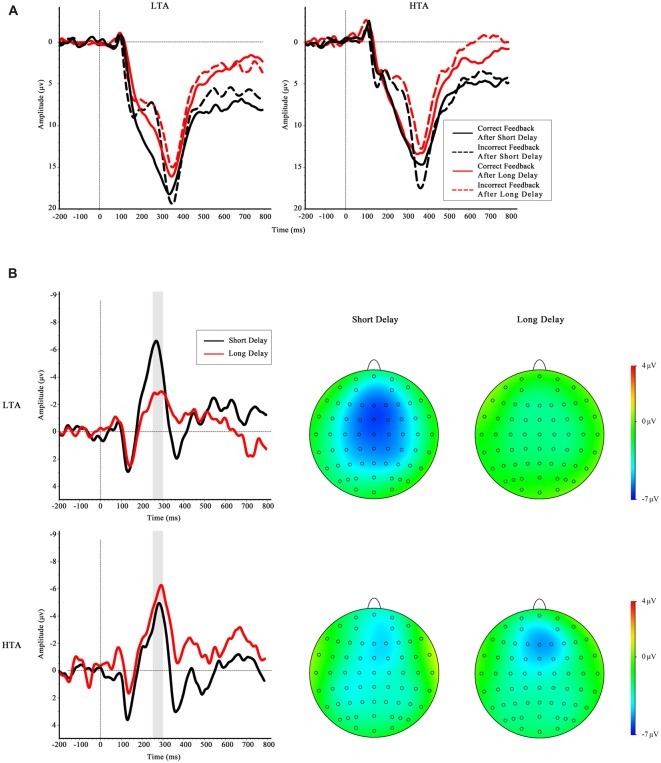
**(A)** Feedback-locked grand-average ERP waveforms at Fz in four conditions for LTA group and HTA group. **(B)** The difference waves of HTA and LTA groups at the Fz. Gray shaded area shows the 250–300 ms analysis window in which the reward positivity (RewP) was quantified.

The grand-average ERPs and RewP for short delay and long delay feedback at Fz are depicted in Figure 5. The main effect of waiting time was not significant, *F*_(1,32)_ = 1.84, *p* = 0.184. The ANOVA yielded significant interactions between trait anxiety group and waiting time, *F*_(1,32)_ = 9.70, *p* = 0.004, $\eta_{\text{p}}^{2}$ = 0.23. A simple effect analysis was performed to investigate the interaction, indicating a greater RewP amplitude in the LTA group when they waited a short time for outcome than when they waited a long time for outcome, *F*_(1,32)_ = 10.0, *p* = 0.003; however, this effect was not found in the HTA group, *F*_(1,32)_ = 1.54, *p* = 0.223. No other significant results were found (all *p* > 0.05).

The ANOVA on P300 amplitude found a reliable main effect of valence, suggesting that the P300 amplitude was greater when the feedback was correct than when the feedback was incorrect, *F*_(1,32)_ = 67.93, *p* < 0.001, $\eta_{\text{p}}^{2}$ = 0.68. The interaction between valence and waiting time was significant, a more positive-going P300 was found when they waited a long time than a short time at the correct feedback condition. In addition, neither the main effect of the trait anxiety group (*F*_(1,32)_ = 0.66, *p* = 0.428) nor the interaction between the group and other factors reached significance: trait anxiety group × valence, *F*_(1,32)_ = 0.39, *p* = 0.538; anxiety group × waiting time, *F*_(1,32)_ = 1.42, *p* = 0.242; trait anxiety group × valence × waiting time, *F*_(1,32)_ = 0.578, *p* = 0.453. To sum up, we did not find any other significant interaction between trait anxiety group and other factors on P300 amplitude. Therefore, it was less likely that the amplitude of the FRN was confounded by P300 in the present results.

## Discussion

In this study, we analyzed the RewP component as an indicator to investigate how waiting time affects outcome evaluation in individuals with high and LTA. To do so, we adopted a novel paradigm, in which a cue informed the participant how long he or she would wait before making a response, and the waiting time before final feedback was manipulated. The results showed that the HTA and LTA individuals had different brain responses to both the cue and feedback stimuli, as reflected by the FRN-like amplitudes and RewP amplitudes, respectively. Specifically, more negative-going FRN-like amplitudes were observed in the long-delay cue condition than in the short-delay cue condition, but only in the HTA group. More importantly, significant differences in RewP amplitude were found between the short- and long-delay conditions in the LTA group, but not in the HTA group.

ERP studies have previously reported some inconsistent findings in regard to feedback processing modulated by waiting time before feedback (Weinberg et al., 2012; Wang et al., 2014; Peterburs et al., 2016; Weismuller and Bellebaum, 2016; Arbel et al., 2017). As we mentioned before, different paradigms and individual traits are two possible reasons for the incompatible results in these studies. Here, we used a modified time-estimation task in which feedback stimuli were mainly dependent on participants’ performance. Compared to the gambling task that we used in our previous study, the present task could provide more information in terms of reinforcement learning.

First, by using a learnable task, the present study could test whether the long delay improved or hampered reinforcement learning efficiency. Consistent with our hypotheses, in a dynamic learning process, waiting time affected learning, which was shown in the absolute change of response time between two adjacent reactions. Several studies have reported better performance for delayed feedback than immediate feedback in scenarios such as vocabulary learning (Metcalfe et al., 2009), geographical representations learning (Guzmán-Muñoz and Johnson, 2008), and learning from a multiple-choice test (Butler et al., 2007). The larger time adjustment in our delayed feedback scenario indicate better performance in changing behavior. Furthermore, the marginal significant interaction of waiting time and group may support the above-mentioned explanations. Compared with the short delayed condition, the long delayed feedback could better promote the learning to adjust participants’ estimation of 1 s only in LTA group. This observations requires further examination in future studies.

In line with previous studies, the waiting time also affected individuals’ brain activities during outcome evaluation (Weinberg et al., 2012; Peterburs et al., 2016; Weismuller and Bellebaum, 2016; Arbel et al., 2017; but see Wang et al., 2014). Weinberg et al. (2012) only found a difference in gains and losses in a short-delay condition, not in a long-delay condition. In contrast, Peterburs et al. (2016) reported a linear effect trend of RewP by waiting time, reflected by gradually less positive-going RewP amplitude with gradually increased waiting time. The observation that delay feedback reduced RewP amplitude were also replicated by two recent studies (Weismuller and Bellebaum, 2016; Arbel et al., 2017). Evidence from EEG and fMRI studies indicates a striatal source following reward and an association between reward feedback ERP signals and the blood oxygenation level dependent response in the ventral striatum (Carlson et al., 2011; Foti et al., 2011; Becker et al., 2014). Nevertheless, the striatum activity involved in feedback processing was reduced when the waiting time changed from short to long (Foerde and Shohamy, 2011). It has recently been proposed that the hippocampus bridges a temporal gap when the learning need to get information across time; with increasing waiting time, the striatum activity in feedback processing is gradually reduced and relies more on the hippocampus for declarative memory (Foerde and Shohamy, 2011). The results of the present study extended these findings by further showing that delay feedback dramatically reduced activity of reward system indexed by RewP amplitude, however, only in LTA group.

Based on the Spielberger (1972) theory, trait anxiety reflects a stable individual difference in the perception of anxiety from context and events. In the current study, HTA individuals exhibited a different processing pattern than the LTA individuals. In contrast to LTA individuals, HTA individuals showed similar RewP amplitudes in the two waiting time conditions. This was in line with attentional control theory, which holds that impaired attentional control makes individuals prioritize the processing of threat stimuli—and the anxiety can be affected not only by the external stimuli examined in most studies, but also by internal stimuli like worry (see review Eysenck et al., 2007). According to the subjective rating in our study, the long delay before feedback caused adverse effects. Psychological entropy theory indicates that individuals need to maintain a balance of entropy, and uncertainty results in high entropy and higher cognitive resource consumption to reach a goal (Hirsh et al., 2012). Thus, the different behavioral and brain response patterns between the two trait anxiety groups in our study may be due to HTA individuals being more sensitive to the imbalance of entropy caused by a long wait before final feedback. Furthermore, both non-human studies (Sapolsky et al., 1985; Uchida et al., 2008) and a human study (Satpute et al., 2012) have found that the distinct subregions of the hippocampus play an important role in mediating the different types of anxiety. Satpute et al. (2012) found that stress caused stronger activity in the dorsal hippocampus for trait anxiety individuals than for normal people. On the other hand, different from state anxiety, trait anxiety activates the posterior hippocampus and posterior cortical region to process the initial appraisal (Fanselow and Dong, 2010). The strong relationship between dorsal hippocampus and anxiety indicated that dorsal hippocampus is not only an important area of learning and memory but also has an association with anxiety. Thus, we speculate that the feedback processing relies more on the hippocampus when waiting time is longer (Arbel et al., 2017), but the trait anxiety individuals prioritize feelings of anxiety and therefore cannot call upon cognitive resources to improve performance through memories. In addition, the behavior results indicated that only the LTA group adjusted their performance differently in the two different waiting time conditions, which is consistent with our view that HTA individuals in the delayed feedback condition cannot effectively use feedback information across time.

In addition to the feedback stage, a larger negative wave was also elicited by the long-delayed cue than by the short-delayed cue. Importantly, this effect was only observed in HTA individuals. Although the cues did not provide feedback information, this FRN-like component appeared in a similar time window as classical FRN and had a similar scalp distribution as the FRN. A few recent studies focused on brain activity in cue and feedback phases, found that delta and theta activities reflected separate processes of these stimuli respectively in decision making (Wang et al., 2016). Not only can feedback modulate our behaviors, but advanced cues can also provide additional information for our subsequent actions. Furthermore, an fMRI study has shown that the anxious individuals exhibited significantly greater intolerance of uncertainty (IU) than the non-anxious individuals (Krain et al., 2008). In Krain et al.’s (2008) study, the anxiety disorders with high IU showed activation of frontal and limbic regions in response to uncertainty. The present finding in cue phase provided electrophysiological evidence that HTA group was more sensitive to the cue of uncertainty condition in terms of long waiting time than LTA group. The HTA individuals might treat long-delay cue as more threat signal, and showed more alertness at the beginning of a long-delay trial. Therefore, compared to the LTA group, HTA individuals maintained higher self-involvement throughout the trials. The relationship between cue-related brain activities and feedback-related activities must be investigated further in future studies.

## Limitation

One limitation of our study is that there was only 17 subjects in each group. Although this sample size is similar to the related published studies in this field (Moser et al., 2008; Dennis and Chen, 2009; Gu et al., 2010a), a relatively larger sample is necessary in future studies, considering the current concern of low replication rate in psychological studies (Button et al., 2013; Open Science Collaboration, 2015).

## Conclusion

In summary, our present study found that waiting time had different influences on the processing of feedback in LTA and HTA individuals. The LTA group reported more happiness in response to correct feedback than the HTA group. Moreover, a marginal interaction effect showed that the LTA group made more adjustments than HTA group. In line with these behavioral data, the present ERP data also found that different brain activity, as indexed by RewP, was observed in the two trait anxiety groups. These results suggested that the HTA group was more sensitive to the anxiety caused by a long delay before feedback and showed similar brain responses during long and short delays. The hyperactivity during waiting time in HTA individuals may hamper flexible behavioral adjustment when they have more time to recall their performance before final feedback.

## Author Contributions

XZ, PL, YL and HL designed this study. XZ and HY performed the study. XZ, HY and PL analyzed the data. YL, XZ, HL and PL wrote the article.

## Conflict of Interest Statement

The authors declare that the research was conducted in the absence of any commercial or financial relationships that could be construed as a potential conflict of interest.

## Footnotes

^1^Note that the RewP component was originally named as FRN in these citations (Weinberg et al., 2012; Peterburs et al., 2016; Weismuller and Bellebaum, 2016; Arbel et al., 2017) in this paragraph.
